# Variations in fatty acid compositions of the seed oil of **Eruca sativa** Mill. caused by different sowing periods and nitrogen forms

**DOI:** 10.4103/0973-1296.71801

**Published:** 2010

**Authors:** Atnan Uğur, İpek Süntar, Sinem Aslan, İlkay Erdoğan Orhan, Murat Kartal, Nazim Şekeroğlu, Dursun Eşiyok, Bilge Şener

**Affiliations:** *Department of Horticulture, Faculty of Agriculture, Ordu University, 52200, Ordu, Turkey*; 1 *Department of Pharmacognosy, Faculty of Pharmacy, Gazi University, 06330, Ankara, Turkey*; 2 *Department of Pharmacognosy, Faculty of Pharmacy, Ankara University, 06110, Ankara, Turkey*; 3 *Department of Biology, Faculty of Art and Science, Kilis 7 Aralik University, 52200 Kilis, Turkey*; 4 *Department of Horticulture, Faculty of Agriculture, Ege University, 35100, Izmir, Turkey*

**Keywords:** Brassicaceae, Eruca sativa, fatty acid, fertilizer, GC-MS, rocket, seed oil

## Abstract

**Background:**

**Eruca** is a native plant genus of the South Europe and central Asia where it has been cultivated since centuries. As the genus name implies, the oil is high in erucic acid.

**Materials and Methods:**

In this study, our aim was to investigate the effect of sowing periods (autumn and spring) and three forms of the nitrogen-containing fertilizers (manure, calcium nitrate [Ca(NO_3_)_2_, 15.5% N], and ammonium sulphate [(NH_4_)_2_SO_4_, 21% N]) on fatty acid compositions of the oils obtained from **Eruca sativa** Mill. seeds cultivated. All oils were obtained by maceration of the seeds with *n*-hexane at room temperature and converted to their methyl ester derivatives by trans-methylesterification reaction using boron-trifluorur (BF_3_). The fatty acid methyl esters (FAMEs) in the oils were detected by capillary gas chromatography-mass spectrometry (GC-MS).

**Results:**

All the samples analyzed were found to contain quite high amounts of erucic acid ranging between 46.64-54.79%, followed by oleic (17.86-19.95%), palmitic (7.25-10.97%), linoleic (4.23-9.72%), and linolenic (1.98-3.01%) acids.

**Conclusion:**

Our data pointed out that there is a statistically important alteration caused by these applications on the contents of only C12:0 and C14:0 found as the minor fatty acids, whereas no other fatty acids in the samples seemed to be affected by those criteria.

## INTRODUCTION

**Eruca sativa** Mill., a member of Brassicaceae family, is commonly referred as “rocket, true rocket, rocket salad, arugula, roquette, or white pepper” in English. The plant, grown in western and southern parts of Turkey about 170– 190 t *per annuum*, is also known as “roka” in Turkey whose leaves are widely consumed for flavoring salads.[1] **Eruca** is a native of southern Europe and central Asia where it has been cultivated since classical times.

**Eruca** seeds contain a large amount of thiofunctionalized glucosinolates along with erucic acid C22:1 (cis-13-docosenoic acid).[2–4] On the other hand, rocket seed oil is used mainly in industry as a lubricant, for soap making, as an illuminating agent, in massage, in medicines, as well as in cooking.[5,6] Furthermore, the oil is quite rich in erucic acid, an important industrial compound, which makes this species a potential industrial crop plant. Besides, erucic acid is sometimes used as adulterant for rapeseed or mustard oils. Relevantly, there is an escalating global demand for the amide of erucic acid, namely erucamide, depending on its use in cosmetics, detergents, and polymer production. Since the crude oil of the plant has a biodegradability property, it is an alternative mineral oil in many industries as mentioned above.[5]

Therefore, a greater attention has been paid to **Eruca sativa** by scientists. For this purpose, we pursued our goal to determine if there could be any effect of sowing times (autumn and spring) and/or various forms of the nitrogen-containing fertilizers including manure, calcium nitrate [Ca(NO_3_)_2_ 15.5% N], and ammonium sulphate [(NH_4_)_2_SO_4_ 21% N] on fatty acid compositions of the seed oils of *E. sativa* samples.

## MATERIALS AND METHODS

### Plant materials and field experimentation

Rocket seed population used in this study was obtained from Faculty of Agriculture, Ege University and field trials were carried out at the experimental areas of Horticultural Department, Faculty of Agriculture, Ege University (Izmir-Turkey) in the 2002-2003 vegetation period. Field experiment was arranged in a split-plot design with three replications. Main plots were sowing times and the subplots were nitrogen forms. At 5 kg ha^−1^ rates and 20-cm row spacing, the rocket seeds were sown by hand in autumn (November) and spring (March) periods. The plot sizes were 2 m^2^. Three different nitrogen forms as manure, calcium nitrate [Ca(NO_3_)_2_ 15.5% N], and ammonium sulphate [(NH_4_)_2_SO_4_ 21% N] with 150 kg ha^−1^ were applied to the plots at sowing time as a factor. Besides nitrogen application, 120 kg ha^−1^ phosphorus (43%, P_2_O_5_) and 180 kg ha^−1^ potassium (K_2_SO_4_, 50%) fertilization were also applied to the all plots prior to sowing. The seeds were harvested at maturity and air-dried at the laboratory. Chemical composition and the other assets of sandy-clay type of the soil in which the *E. sativa* seeds were cultivated are given in Table 1.

**Table 1:** Chemical composition and the other properties of the experimental area soils

pH	Salt (%)	CaCO3 (%)	Organic matter (%)	N (%)	P (ppm)	K (ppm)	Ca (ppm)	Mg (ppm)	Fe (ppm)	Cu (ppm)	Zn (ppm)	Mn (ppm)
7.36	0.059	3.6	2.06	0.1	4.2	460	3750	56	52	4.6	0.9	26

### Extraction of the oils

The seed samples of *E. sativa* used in this study were ground in a grinder in the presence of anhydrous sodium sulphate and accurately weighed. The ground plant materials (approximately 10 g for each) were macerated with 300 ml of *n*-hexane at room temperature for 2 days, the macerates were shaken occasionally. Following filtration of the organic phases, the *n*-hexane phases of each sample was concentrated in vacuo at 40°C by evaporating and concentrated oils were obtained.

### Methyl esterification of the fatty acids

Three replicates comprising of healthy looking seeds were analyzed. The oils were independently placed in 25 ml of volumetric flasks, then saponified by adding 12 ml 0.5 N methanolic sodium hydroxide to each mixture, and were heated on a steam bath until the fat globules disappeared. 2 ml of BF_3_ /MeOH (Sigma Co.) was added to each flask and the mixtures were boiled for 2 minutes. After the solutions were cooled down at room temperature, they were filled up to 25 ml with saturated sodium chloride solution and the fatty acid methyl esters (FAMEs) were prepared for each sample.[7] The obtained FAMEs were dissolved in *n*-hexane and 1 μl of samples was injected and analyzed by GC-MS.

### GC-MS analysis conditions

Chromatographic analysis by GC-MS analysis was carried out on Agilent 6890N Network GC system combined with Agilent 5973 Network Mass Selective Detector (GC-MS). The capillary column used was an Agilent 19091N-136 (HP Innowax Capillary; 60.0 m × 0.25 mm × 0.25 μm). Helium was used as carrier gas at a flow rate of 3.3 ml/min with 1 μl injection volume. Samples were analyzed with the column held initially at 100°C for 1 min after injection, then increased to 170°C with 10°C/min heating ramp without hold and increased to 215°C with 5 °C/min heating ramp for 5 min. Then final temperature was increased to 240°C with 10°C/min heating ramp for 10.5 min. The injection was performed in split mode (20:1) at 270 °C. Detector and injector temperatures were 280°C and 250°C, respectively. Run time was 35 min. MS scan range was (*m/z*): 35-450 atomic mass units (AMU) under electron impact (EI) ionization (70 eV).

### Identification of the fatty acids

Fatty acid components of the oils of eighteen different *E. sativa* seeds were determined by comparing their retention times to authentic fatty acid samples obtained by GC and mass fragmentations with those of mass spectra database search (Wiley and Nist). They were also compared to mass spectrums of authentic fatty acid standards with GC-MS. Authentic standard used in this study was FAME mix Supelco-1891-1AMP [containing palmitic acid methyl ester 16:0, stearic acid methyl ester 18:0, oleic acid methyl ester 18:1, linoleic acid methyl ester (18:0; cis 9,12), linolenic acid methyl ester (18:3; cis 9,12,15)], and arachidonic acid methyl ester (20:4) (Sigma-A9298).

### Statistical analysis

The data were subjected to analysis of variance (ANOVA) following the general linear models with sowing periods and nitrogen forms as the main treatment effects. Treatment means were separated using least significant differences expressed as LSD at 5% of probability. All statistical analysis was carried out using the MSTATC computer software.

## RESULTS AND DISCUSSION

In our study, the fatty acid profiles of the eighteen seed samples conformed to the patterns described in the literature for rocket oils with the proportions of different fatty acids fitting within the reported ranges[8–12] except for one study done by Flanders and Abdulkarim.[13] The results on our samples confirmed that erucic acid was the major component comprising of 46.64– 54.79%, followed by palmitic (7.25– 10.97%), oleic (17.86– 19.95%), linoleic (4.23– 9.72%), and linolenic (1.98– 3.01%) acids [Table 2].

**Table 2:** Variations in fatty acid compositions (%) of the rocket seed oils by sowing times

Fatty acids	Sowing periods		LSD (5%)
	Autumn	Spring	
C8:0	0.21	0.15	NS
C11:0	0.07	0.00	NS
C12:0	0.10	0.00	0.064
C6:0	0.12	0.20	NS
C14:0	0.13	0.08	0.020
C16:0	8.07	10.97	NS
C16:1	0.14	0.16	NS
C18:0	2.38	2.28	NS
C18:1 (n9c)	18.28	19.09	NS
C18:2 (n6c)	4.23	9.72	NS
C18:3 (n3)	2.07	3.01	NS
C20:0	0.75	0.42	NS
C20:1	8.12	7.19	NS
C22:0	0.22	0.08	NS
C22:1 (n9)	54.79	46.64	NS
Total	99.68	99.99	NS

NS: Not Significant

In this study, we aimed to establish whether there is any relationship between sowing times (autumn and spring) and three forms of the nitrogen-containing fertilizers; namely, manure, calcium nitrate [Ca(NO_3_)_2_ 15.5% N], and ammonium sulphate [(NH_4_)_2_SO_4_ 21% N)], on fatty acid compositions of the eighteen seed oils obtained from *E. sativa* samples. Our data pointed out that there is a statistically important alteration caused by these applications on the contents of only C12:0 and C14:0 fatty acids, whereas no other fatty acids in the samples seemed to be affected by those criteria, statistically [Tables 2 and 3]. Concerning the sowing periods, nine of the fatty acids were observed to reach at their highest levels in autumn time, while six of them attained to their highest levels in spring. Furthermore, we also figured out that the amount of erucic acid was found to be higher when sowed in autumn. Taking various types of nitrogenous fertilizers into account, no statistically significant variation was observed in fatty acid compositions, although ten out of fifteen fatty acids detected herein were higher in the manure-applied samples [Table 3]. There was no significant variation in erucic acid (C22:1, n9) content for all analyzed treatment effects.

**Table 3:** Variations in fatty acid compositions (%) of the rocket seed oils by nitrogen forms

Fatty acids	Fertilizer types		
	Manure	Ammonium	Nitrate
C8:0	0.19	0.20	0.16
C11:0	0.05	0.04	0.01
C12:0	0.07	0.06	0.02
C6:0	0.06	0.23	0.19
C14:0	0.16	0.09	0.07
C16:0	7.25	10.76	10.54
C16:1	0.23	0.08	0.14
C18:0	2.37	2.27	2.34
C18:1(n9c)	19.95	18.26	17.86
C18:2 (n6c)	7.93	6.19	6.80
C18:3 (n3)	2.69	1.98	2.96
C20:0	0.84	0.50	0.42
C20:1	8.58	7.33	7.06
C22:0	0.24	0.23	0.00
C22:1 (n9)	49.38	51.77	51.01
Total	99.99	99.99	99.58

NS: Not Significant

In accordance with our current data, some other researchers previously reported that fatty acid composition could change depending on different sowing periods in oil crops.[14,15] Besides, the fatty acid composition in oil crops is also changeable due to their maturing periods, which is explained by a theory that synthesis of unsaturated fatty acids diminish, while the amount of saturated fatty acids boost.[16,17] In a recent study published by Şekeroğlu and Özgüven,[18] it was stated that the decrease in γ-linolenic acid quantity in *Oenothera biennis* L. was due to higher temperature (over 30°C) in Çukurova region of Turkey. Ahmad and Abdin[19] also stated that nitrogen probably promotes elongation of the carbon chain of rapeseed oil; however, fatty acid composition changes by nitrogen.

In conclusion, outcomes of this study reported here will be a beneficial point for future breeding in order to improve yield and quality of the rocket oil. To the best of our knowledge, this is the first study hitherto investigating the effects of different sowing periods and fertilizer types on fatty acid compositions of the seed oils of *E. sativa* samples. However, it is also suggested to investigate the influences of different environmental factors in multi-location trials.

## References

[1] Esiyok D, Padulosi S, Pignone D (1997). Marketing and utilization of rocket in turkey. Rocket: A mediterranean crop for the world.

[2] Bennett RN, Mellon FA, Botting NP, Eagles J, Rosa EA, Williamson G (2002). Identification of the major glucosinolate (4-mercaptobutyl glucosinolate) in leaves of **Eruca sativa** l.(salad rocket). Phytochemistry.

[3] Falk KL, Vogel C, Textor S, Bartram S, Hick A, Pickett JA (2004). Glucosinolate biosynthesis: Demonstration and characterization of the condensing enzyme of the chain elongation cycle in **Eruca sativa**. Phytochemistry.

[4] Lazzeri L, Errani M, Leoni O, Venturi G (2004). **Eruca sativa** spp.*Oleifera*: A new non-food crop. Ind Crop Pro.

[5] Ahh A, Khalil MK, Farrag AM (2002). Nitrate accumulation, growth, yield, and chemical composition of rocket (**Eruca sativa**) plant as affected by npk fertilization, kinetin and salicylic acid. Ann Agric Sci (Cairo).

[6] Miyazawa M, Maehara T, Kurose K (2002). Composition of the essential oil from the leaves of **Eruca sativa**. Flav Frag J.

[7] Morrison WR, Smith LM (1964). Preparation of fatty acid methyl esters and dimethylacetals from lipids with boron trifluoride-methanol. J Lipid Res.

[8] Padulosi S, Pignone D (1997). A mediterranean crop for the world.

[9] Yadava TP, Friedt DW, Gupta SK (1998). Oil content and fatty acid composition of taramira (**Eruca sativa**) genotypes. J Food Sci Tech.

[10] Das S, Tyagi AK, Singhal KK (2001). Chemical composition including amino acid, fatty acid, and glucosinolate profile of taramira (**Eruca sativa**) oilseed. Ind J Agric Sci.

[11] Mandal S, Yadav S, Singh R, Begum G, Suneja P, Singh M (2002). Correlation studies on oil content and fatty acid profile of some cruciferous species. Gen Res Crop Evo.

[12] Zanetti F, Vamerali T, Bona S, Mosca G (2006). Can we “cultivate” erucic acid in southern europe?. Int J Agr.

[13] Flanders A, Abdulkarim SM (1985). Composition of seed and seed oils of taramira (**Eruca sativa**). J Am Chem Soc.

[14] Sang JP, Bluett CA, Elliott BR, Truscott RJ (1986). Effect of time of sowing on oil content, erucic acid and glucosinolate contents in rapeseed (*Brassica napus* l. Cv. Marnoo). Aus J Exp Agric.

[15] Pritchard FM, Eagles HA, Norton RM, Salisbury PA, Nicolas M (2000). Environmental effects on seed composition of victorian canola. Aus J Exp Agric.

[16] Lotti G, Izzo R, Bottazzi F, Bellani A (1984). Influence of sowing date on the development cycle and plant characteristics of *Oenothera biennis* L. Int Q Agric Sci.

[17] Levy A, Yaniv Z, Menagem E, Barzilai M (2002). Genotype-temperature interaction for gamma-linolenic acid content in seeds of *Oenothera lamarckiana l*.Proceedings of the workshop on agricultural and quality aspects of medicinal and aromatic plants.

[18] Şekeroğlu N, Özgüven M (2006). Effects of different nitrogen doses and plant densities on yield and quality of *Oenothera biennis* l. Grown in irrigated lowland and un-irrigated dryland conditions. Tr J Agric Forest.

[19] Ahmad A, Abdin MZ (2000). Interactive effect of sulphur and nitrogen on the oil and protein contents and on the fatty acid profiles of oil in the seeds of rapeseed (*Brassica campestris* l.) and mustard (*Brassica juncea* l. Czern.

